# In this month’s Bulletin

**DOI:** 10.2471/BLT.21.001021

**Published:** 2021-10-01

In the editorial section, Alarcos Cieza et al. (686) encourage submissions to a theme issue on health policy and systems research for rehabilitation. 

Andrey Shukshin (689–690) reports that tobacco control legislation is helping curb tobacco use in the Russian Federation. Agnes Kalibata talks to Gary Humphreys (691–62) about the need for multisectoral food system reform.

## Australia, New Zealand

### COVID-19 vaccines for pregnant and lactating women

Michelle L Giles et al. (739–746) provide a case study on alignment of national recommendations.

## Barbados, Belize, Jamaica, Saint Vincent and the Grenadines

### Systems science for policy 

Leonor Guariguata et al. (722–729) explore ways to address declining levels of physical activity.

## Brazil, China, Colombia, Dominican Republic, Ecuador, Egypt, Ghana, Honduras, India, Islamic Republic of Iran, Iraq, Kenya, Lao Peoples’ Democratic Republic, Malaysia, Namibia, Nicaragua, Pakistan, Papua New Guinea, Paraguay, Peru, South Africa, Suriname, Timor-Leste, United Republic of Tanzania

### Preventing maternal deaths in middle-income countries 

Anke Heitkamp et al. (693–707) review the evidence for near-miss events.

## Brazil, Israel, Spain, United States of America 

### Efficiency at scale

Marie Reilly & Bhavna Chohan (708–714) describe pooled testing options for SARS-CoV-2.

## European Region

### Collaborating for improved access

Sabine Vogler et al. (715–721) detail joint medicine and vaccine procurement arrangements. 

## Global

### Unintended effects of COVID-19 restrictions

Amiya Bhatia et al. (730–738) argue for better prevention of violence against children.

### Measuring equity of access to family planning

Sara Stratton et al. (747–749) propose a move beyond wealth status and contraceptive use.

### Save the planet

Lukoye Atwoli et al. (750–752) call for action to limit global temperature increases, restore biodiversity and protect health.

